# Predicting non-small-volume central lymph node metastases (>5 or ≥2 mm) preoperatively in cN0 papillary thyroid microcarcinoma without extrathyroidal extension

**DOI:** 10.1097/MD.0000000000022338

**Published:** 2020-09-18

**Authors:** Jin-Duo Shou, Fei-Bo Li, Liu-Hong Shi, Liang Zhou, Lei Xie, Jian-Biao Wang

**Affiliations:** a Departments of Diagnostic Ultrasound and Echocardiography, the Affiliated Sir Run Run Shaw Hospital, Zhejiang University School of Medicine, Hangzhou; b Second Department of General Surgery, Zhejiang Putuo Hospital, Zhoushan; c Head and Neck Surgery, the Affiliated Sir Run Run Shaw Hospital, Zhejiang University School of Medicine, Hangzhou, Zhejiang, P. R. China.

**Keywords:** central lymph node metastasis, non-small-volume central lymph node metastases, papillary thyroid microcarcinoma, predictive factors, thyroid surgery

## Abstract

The ability to identify patients with aggressive papillary thyroid microcarcinoma (PTMC) from the low-risk patients is critical to planning proper management of PTMC. Lymph node metastases showed association with recurrence and low survival rate, especially in patients with >5 or ≥2 mm metastatic lymph nodes. Therefore, this study aimed to investigate the preoperatively predictive factors of non-small-volume (metastatic lymph nodes >5 or ≥2 mm in size) central lymph node metastases (NSVCLNM) in PTMC patients. A total of 420 patients with clinically node-negative (cN0) PTMC without extrathyroidal extension underwent thyroidectomy plus central neck dissection (CND) between January 2013 and December 2015, were retrospectively analyzed. Of the 420 patients, 33 (7.9%) had NSVCLNM. The 5-year recurrence-free survival was significantly less in cN0 PTMC patients with NSVCLNM, when compared with patients without NSVCLNM (80.8% vs 100%, *P* < .001). Multivariate logistic regression revealed age ≤36 years (*P* < .001), male sex (*P* = .002), ultrasonic tumor sizes of >0.65 cm (*P* < .001), and ultrasonic multifocality (*P* = .039) were independent predictive factors of NSVCLNM. A prediction equation (Y = 1.714 × age + 1.361 × sex + 1.639 × tumor size + 0.842 × multifocality −5.196) was developed, with a sensitivity (69.7%) and a specificity (84.0%), respectively, at an optimal cutoff point of −2.418. In conclusion, if the predictive value was >−2.418 according to the equation, immediate surgery including CND rather than active surveillance might be considered for cN0 PTMC patients.

## 1 Introduction

Surgery is the first-line therapy for papillary thyroid carcinoma (PTC). However, surgery is associated with the risk of complications and the need for levothyroxine treatment.^[1]^ Although there is general consensus that benefit outweighs risk for surgery performed for clinically significant PTC >1 cm,^[2]^ there is a growing body of evidence suggesting that active surveillance potentially can serve as a safe alternative to immediate surgery for at least papillary thyroid microcarcinoma (PTMC).^[3–6]^ However, a small percentage of patients with PTMC still would develop clinically significant regional or distant metastases or cancer-related death.^[7–10]^ Unfortunately, no clinical features^[2]^ can reliably differentiate the relatively small number of PTMC patients destined to develop clinically significant progression from the larger population of people that harbor indolent PTMCs that will not cause significant disease.

Cervical lymph node metastasis is quite common in patients with PTMC. Even for clinically node-negative (cN0) PTMC, central lymph node metastasis (CLNM) is found in 60.9% cases.^[11]^ Lymph node metastasis of PTMC is known to correlate with higher local recurrence,^[12–15]^ lower overall survival rate,^[9]^ and shorter disease-specific survival^[16]^, especially for patients with ≥5 metastatic lymph nodes^[10,17]^ or metastatic lymph node ≥2 mm.^[15,18]^ According to American Thyroid Association (ATA) 2015 Risk of recurrence Stratification System, PTMC patients with >5 or ≥2 mm (in largest dimension) pathological metastatic lymph nodes are not in the low risk.^[2]^ Cho et al have reported that the 5-year recurrence-free survival (RFS) for PTMC patients with metastatic lymph node ≥2 mm is less compared with patients without metastasis or metastatic lymph node <2 mm (89.2% vs 96.8%, *P* = .02).^[15]^ In addition, Sugitani et al have found that the risk of recurrence is significantly higher in PTC patients with ≥5 lymph node metastases (19%) compared to those having <5 metastases (8%).^[17]^ Therefore, PTMC patients harboring >5 or ≥2 mm metastatic lymph nodes in the central compartment are of less favorable outcome.

Hence, in this study, the incidence and preoperatively predictive factors for >5 or ≥2 mm central lymph node metastases (defined as non-small-volume central lymph node metastases, NSVCLNM) in a group of cN0 PTMC patients without extrathyroidal extension (ETE) were investigated. A prediction model was then developed to estimate the risk of NSVCLNM and facilitate therapeutic decision for PTMC patients.

## 2 Materials and methods

### 2.1 Patients

In total, 420 cN0 PTMC patients without ETE and underwent total thyroidectomy or lobectomy plus prophylactic central neck dissection (CND) between January 2013 and December 2015 were included in this retrospective study. The medical records of these patients were retrospectively reviewed based on a prospectively collected database from the Department of Head and Neck Surgery in the Affiliated Sir Run Run Shaw Hospital, Zhejiang University School of Medicine. The exclusion criteria were as follows: patients with non-PTCs (follicular/medullary/anaplastic cancer), tumor size >1 cm, ETE, clinically apparent metastatic disease to nodes (cN1), distant metastasis, or with a history of previous thyroid surgery. A cN1 neck was defined as patients suspected with lymph node metastasis in central or lateral neck by ultrasonography (US) and computed tomography (CT) or proved by ultrasound-guided fine needle aspiration (FNA) preoperatively. The state of ETE was evaluated based on intraoperative findings and paraffin section pathological examination.

Thyroid nodules, as well as central and lateral neck lymph nodes, were evaluated using US examination in each patient. FNA was then used to confirm the malignancy or rule out metastasis of suspicious primary lesions in the lateral cervical lymph nodes. Vocal cord function was assessed by direct or indirect laryngoscopy. In addition, the levels of thyroid hormone, thyroid peroxidase antibody, thyroglobulin antibody, parathyroid hormone, calcitonin, and serum calcium were also measured in each patient.

This study was approved by the Ethical Committee of the Affiliated Sir Run Run Shaw Hospital, Zhejiang University School of Medicine.

In accordance with the guidelines of ATA (2009),^[19]^ total thyroidectomy was performed if the patient met one of the following criteria: bilateral nodularity, ETE, tumor diameter >1.0 cm, multifocal lesions in the affected lobe, and regional or distant metastases. Thyroid lobectomy was performed for small (≤1 cm), unifocal and intrathyroidal papillary carcinomas in the absence of previous head and neck irradiation, or radiologically or clinically involved cervical node metastases. Ipsilateral prophylactic central neck dissection (pCND) was performed routinely, whereas bilateral pCND was performed if the lesions occurred in the isthmus or the malignant nodules were located in both lobes.

### 2.2 CND surgical procedure

All the procedures included were conventional open surgeries and were performed by 3 senior surgeons. Thyroidectomy was performed by using the technique of capsular dissection as suggested by Thompson et al.^[20]^ Recurrent laryngeal nerves and all parathyroid glands were routinely identified and preserved under direct vision. The vascular supply of the parathyroid glands was confirmed by fine needle pricking test. Devascularized parathyroid gland was excised into tiny fragments and was autotransplanted into the contralateral sternocleidomastoid muscle. Based on ATA guidelines, bilateral CND entails removal of the prelaryngeal, pretracheal, and both the right and left paratracheal nodal basins; unilateral CND involves removal of the prelaryngeal, pretracheal, and the single paratracheal nodal basin.^[21]^

### 2.3 Sonographic analysis and pathology

US inspection was performed by 4 diagnostic medical sonographers with >5 years of experience. The following US findings associated with NSVCLNM were documented: multifocality, tumor size, calcification (including microcalcifications and macrocalcifications), irregular margin (defined as either infiltrative, microlobulated, or spiculated), and vascularity (rich blood flow, little blood flow, or no flow).

In this study, the central compartment lymph nodes were collected and analyzed. The number of metastatic lymph nodes was based retrospectively on the initial pathological reports. Specifically, all metastatic foci in lymph nodes were microscopically measured by 1 pathologist who prospectively reviewed the slides of all metastatic lymph nodes. In cases wherein multiple metastatic foci were observed, the largest one was recorded as the size of metastatic lymph node. The NSVCLNM was defined as metastatic lymph node >5 or ≥2 mm in size, located in the central neck compartment.

### 2.4 Follow-up

Before 2016, the treatment protocol for PTMC in our hospital was slightly aggressive. Radioactive iodine therapy was routinely suggested for all patients with differentiated thyroid carcinoma if total thyroidectomy was performed, unless the patient refused. Therefore, most patients who underwent total thyroidectomy in this study were administrated with radioactive iodine therapy, whether having lymph node metastasis or not. The dose of radioactive iodine therapy for cN0 PTMC patients without ETE in our center was major 100 mCi, regardless of the state of lymph node metastases.

All patients received thyroid-stimulating hormone-suppressive therapy after surgery or radioactive iodine therapy. US examinations of neck were performed every 3 to 6 month for the first 2 years after surgery and every 12 month for rest years. The levels of thyroglobulin and thyroglobulin antibody were routinely measured.

Recurrence was defined as the presence of clinical or radiologic evidence of disease on review of medical record and imaging findings, and confirmed pathologically with FNA or operation, >6 months after primary thyroid surgery.

### 2.5 Statistical analysis

Statistical analyses were performed using SPSS version 16.0 (IBM, Armonk, New Y). Continuous data are present as mean (s.d.) or median (range). Baseline patient characteristics were compared between the groups using Student *t* test or Mann-Whitney *U* test for continuous variables as appropriate, and Pearson *χ*^2^ test for categorical variables. A receiver-operating characteristic (ROC) analysis was used to identify the cutoff point of the primary tumor size, age, and Y value for defining the risk of NSVCLNM. The odds ratio (OR) and 95% confidence interval (CI) for relationships between each variable and NSVCLNM (yes/no) were calculated using binary logistic regression. RFS was defined as the time interval between the date of thyroid surgery and the date of recurrence. RFS curves were plotted using Kaplan-Meier method and compared using the log-rank test. All tests were 2-sided and a *P* < .05 was defined to be statistically significant.

## 3 Results

### 3.1 Patient characteristics

In total, 420 patients with cN0 PTMC without ETE confirmed by postoperative pathology were included in this study. A total of 111 patients involved with ultrasonic multifocal lesions were all confirmed by postoperative pathology. The median number of harvested central lymph nodes was 11.0 (range 1–43), and the median number of metastatic central lymph nodes was 2.0 (range 1–17). CLNM was detected in 121 (28.8%) patients, whereas NSVCLNM was detected in 33 (7.9%) patients. The median size of largest metastatic foci in the central lymph nodes from 121 patients with CLNM was 1.13 mm (0.06 mm−5.62 mm), and 76.9% (93 of 121) of the metastatic foci were <2 mm. Of all the 420 PTMC patients, 333 (79.3%) were women and 87 (20.7%) were men, with a mean age of 44.0 years (range, 19–78 years), and 80 (19.0%) were ≥55 years. Overall, 3 different surgical procedures were performed: lobectomy (including isthmectomy and pyramidal lobectomy) plus ipsilateral pCND (right 103 patients, and left 92 patients); total thyroidectomy plus ipsilateral pCND (right 65 patients, and left 68 patients); and total thyroidectomy plus bilateral pCND (92 patients).

No patients presented with any history of head and neck radiation before diagnosis, and none had distant metastasis. The characteristics of these patients are listed in Table 1.

**Table 1:**
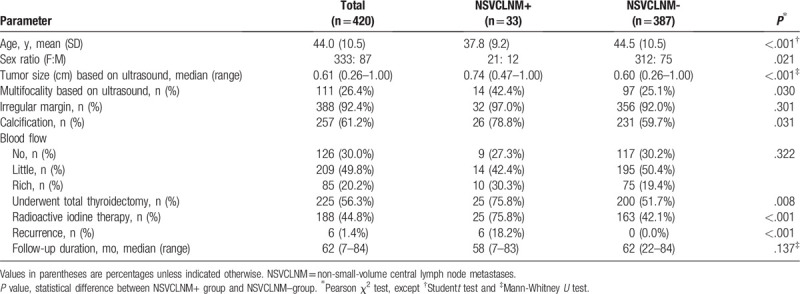
Patient characteristics.

### 3.2 Predictive factors for NSVCLNM in cN0 PTMC patients without ETE

**Figure 1:**
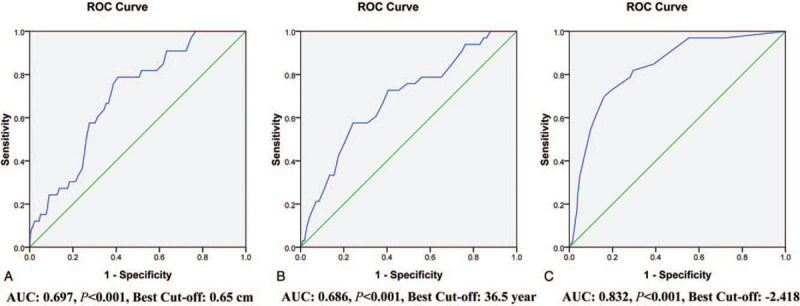
The ROC curve analyses of cut-off point for size of the primary tumor (A), age (B), and Y value (C) in predicting non-small-volume central lymph node metastasis in clinically node-negative papillary thyroid microcarcinoma. AUC = area under the curve, ROC = receiver-operating characteristic.

We used ROC curve analysis to determine the cutoff point for size of the primary tumor (0.26–1.0 cm) in predicting NSVCLNM. An ultrasonic tumor size of 0.65 cm was the cutoff point for NSVCLNM in our study (Fig. 1A). Tumor size >0.65 cm was significantly associated with NSVCLNM in univariate logistic regression analyses (Table 2).

**Table 2:**
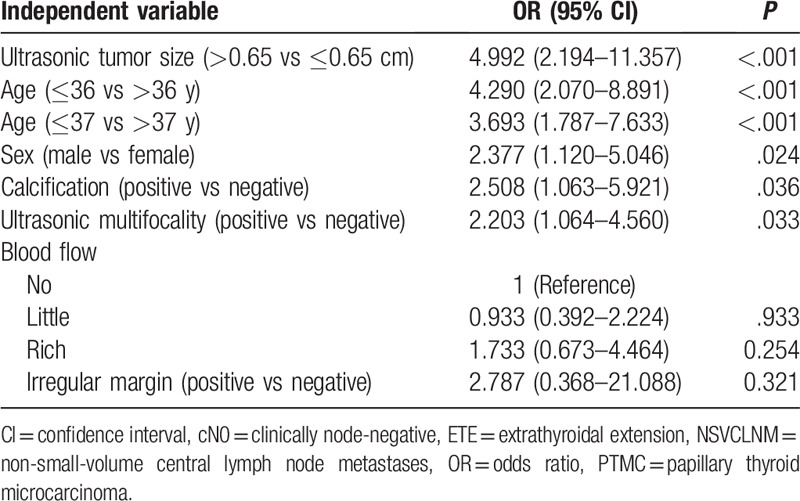
Univariate logistic regression analyses of NSVCLNM in cN0 PTMC patients without ETE.

Additionally, NSVCLNM more likely occurs among young patients (Table 1). Consequently, ROC curve analysis was used to determine the cut-off point of age (range, 19–78 years) for predicting NSVCLNM in patients with cN0 PTMC, and found that the cut-off point of age to be 36.5 years (Fig. 1B). Ages ≤36 years and ≤37years were both significantly associated with NSVCLNM according to the univariate logistic regression, but the former had a higher OR and 95% CI (Table 2).

Univariate analyses also revealed that male sex, calcification, and multifocal lesions identified by US were significantly associated with NSVCLNM in cN0 PTMC patients. Other factors, including blood supply and irregular margin of the tumor, were not significantly related to NSVCLNM.

Multivariate logistic regression analyses confirmed that age ≤36 years, male sex, ultrasonic tumor size >0.65 cm, and ultrasonic multifocality, were independent predictive factors of NSVCLNM in cN0 PTMC patients without ETE (Table 3). The predictive efficiency of each independent predictive factor was listed in Table 4.

**Table 3:**
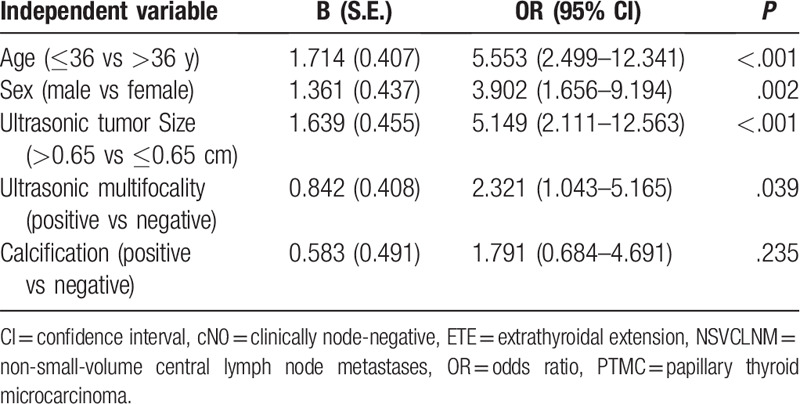
Multivariate logistic regression analyses of NSVCLNM in cN0 PTMC patients without ETE.

**Table 4:**
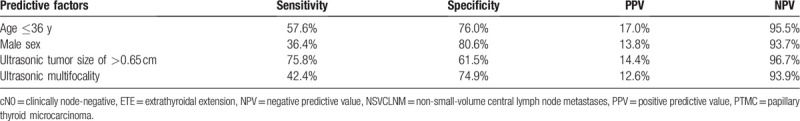
Predictive efficiency of each independent predictive factor for NSVCLNM in cN0 PTMC patients without ETE.

### 3.3 Prediction model of NSVCLNM in cN0 PTMC patients without ETE

Using the coefficients (B) obtained from multivariate logistic regression analyses, the following prediction equation for NSVCLNM was derived, in which the categorical variables were coded as “1” if present (younger than 36 years, male sex, tumor size of >0.65 cm, and multifocality) and “0” if absent:

Y = 1.714 × age + 1.361 × sex + 1.639 × tumor size + 0.842 × multifocality −5.196, where Y means the chance of a positive of NSVCLNM.

Consequently, the ROC curve analysis was used to determine the optimal cut-off point of Y (range, −5.200 to 0.360) for predicting NSVCLM in cN0 PTMC patients without ETE (Fig. 1C). The optimal cutoff point was selected as −2.418, which meant that patients with a predictive value of >−2.418 had the maximum likelihood to involve with NSVCLNM. The sensitivity, specificity, positive predictive value (PPV), and negative predictive value (NPV) were 69.7%, 84.0%, 27.1%, and 97.0%, respectively. The Hosmer-Lemeshow test showed no significance (*P* = .518), indicating a good fit of the model.

### 3.4 Follow-up and recurrence

**Figure 2:**
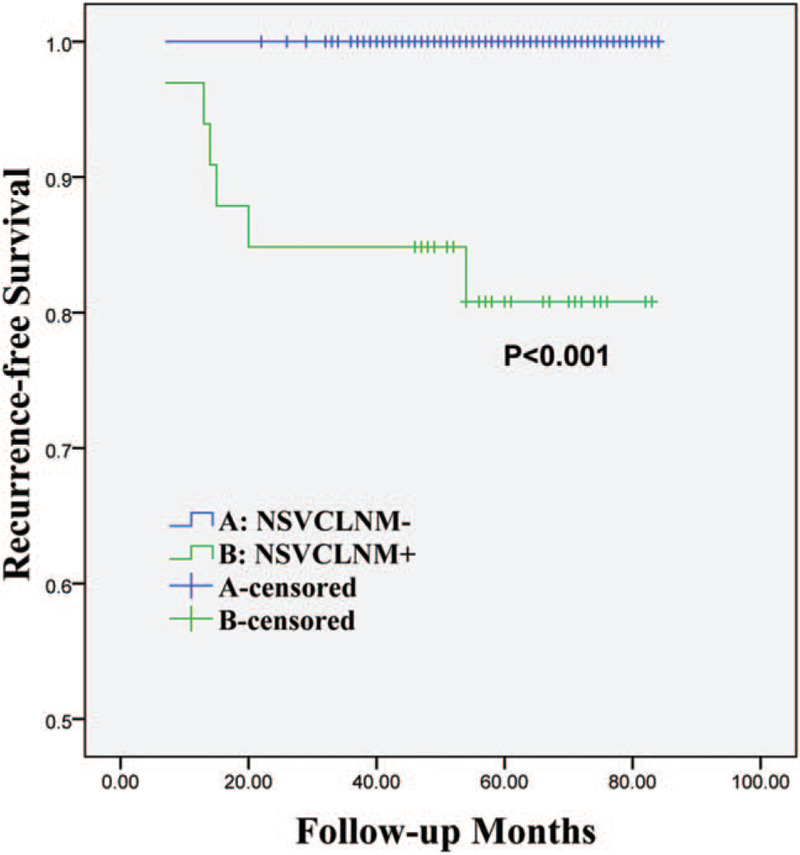
Kaplan-Meier curves of recurrence-free survival in 420 patients with clinically node-negative papillary thyroid microcarcinoma according to NSVCLNM. NSVCLNM = non-small-volume central lymph node metastasis.

The median follow-up duration was 62 (7–84) months. During the follow-up period, 6 PTMCs (1.4%) recurred in the neck lymph node or remnant thyroid. The recurrence was confirmed pathologically with operation in all patients. Kaplan-Meier RFS curves showed that the 5-year RFS for patients with NSVCLNM was significantly less, when compared with patients without NSVCLNM (80.8% vs 100%, *P* < 0.001) (Fig. 2). Radioactive iodine therapy was administrated to more patients with NSVCLNM, when compared with those patients without NSVCLNM (75.8% vs. 42.1%, *P* < .001). Interestingly, there was no recurrence in patients with NSVCLNM negative.

## 4 Discussion

Controversy exists over optimal management of PTMC. The authors of 2015 ATA guidelines note that, although surgery is generally recommended for cytologically confirmed thyroid cancer, an active surveillance management approach “can be considered” as an alternative to immediate surgery in patients with PTMC without clinically evident metastases or local invasion, and no convincing cytologic evidence of aggressive disease.^[2]^ A prospective observational trial of PTMC was initiated in 1993 at Kuma Hospital,^[3,22]^ Kobe, Japan, and a similar trial started at the Cancer Institute Hospital in Tokyo in 1995.^[4,23]^ In these protocols, patients with thyroid nodules measuring ≤1 cm confirmed to be PTC by FNA biopsy were allowed to choose between 2 alternative treatment options—immediate surgery or active surveillance.^[5]^ By 2014, Miyauchi et al from Kuma Hospital had enrolled 1235 patients in the active surveillance program, and showed that 8.0% of PTMC had a size enlargement of >3 mm and 3.8% were found to have novel regional nodal metastases after a 10-year observation period.^[3]^ A series of 230 patients with 300 PTMCs from The Cancer Institute Hospital Tokyo were enrolled in the active surveillance program.^[4]^ With a mean follow-up of 5 years (range, 1–17 years), tumor enlargement of >3 mm was identified in 7.3% of lesions, and 1.3% of patients developed lateral nodal metastases.^[4]^

PTMC typically has an indolent behavior with a good prognosis but it is not always completely harmless. Following thyroid surgery for PTMC, disease-specific mortality rates have been reported up to 1%, locoregional recurrence rates are 2% to 6%, and distant recurrence rates are 1% to 2%.^[7–10,24]^ In addition, Nilublo et al reported PTC ≤2 cm accounted for 12.3% of patients with thyroid cancer-related mortality, and PTMC accounted for 5.1% of patients with thyroid cancer-related mortality.^[16]^ Therefore, Nilublo et al suggested nonoperative management for patients with PTC ≤2 cm should be used with caution.

At present, the value of pCND for cN0 PTC remains unclear, especially in PTMC. Nonetheless, some countries such as China and Japan underline the significance of the routine pCND for cN0 PTMC,^[25]^ which can not only reduce local recurrence rate, but also identify the stage of disease to guide subsequent treatment such as thyroid-stimulating hormone-suppression and radioactive iodine treatment.^[26]^ A meta-analysis enrolled in 17 studies observed the locoregional recurrence of PTC patients after surgery, showed that pCND reduced the risk of lymph node recurrence by 34%.^[27]^ Barczyński et al also showed the 10-year local recurrence rates of patients with and without pCND were 5.6% and 13.4%, respectively.^[28]^ Popadich et al found that pCND in cN0 PTC is associated with lower postoperative Tg levels and reduces the need for reoperation in the central compartment.^[29]^ So, it is interesting to identify the subsets of PTMC patients who will benefit from pCND.

The ability to identify patients with aggressive PTMCs from the majority of low-risk patients is critical to planning proper clinical management. Unfortunately, no clinical features^[2]^ can reliably differentiate the aggressive PTMC patients from the indolent ones. Although there is much interest in using molecular testing to guide the management of PTMC, no test to date has been developed that can accurately predict clinical outcomes in PTMC.^[30]^ Although the BRAF V600E mutation conveys an increased risk in intermediate- and high-risk patients, it does not appear to identify those PTMC at risk of disease progression.^[31–33]^

The correlation between lymph node metastases and tumor recurrence in PTMC has been confirmed in different aspects by many studies.^[12–15]^ Moreover, Nilublo et al have reported lymph node metastases were the independent risk factors for cancer-related death from PTC ≤2 cm.^[16]^ The study conducted by Yu et al showed that PTMC patients with lymph node metastases had poorer overall survival.^[9]^ In addition, According to current ATA 2015 Risk of recurrence Stratification System, PTMC patients with >5 or ≥2 mm metastatic lymph nodes are not in the low risk.^[2]^ In this study, we also found PTMC patients with NSVCLNM had significantly poorer 5-year RFS. Therefore, PTMC patients harboring CLNM, especially NSVCLNM, are absolutely of less favorable prognosis. Better knowledge regarding the preoperatively predictive factors of NSVCLNM from this study suggested a more selective approach to identify cN0 PTMC patients with the necessity of immediate surgery rather than active surveillance.

In this study, we have systematically analyzed the frequency and preoperatively predictive factors of NSVCLNM in 420 cN0 PTMC patients without ETE. To our knowledge, this is the first study concerning NSVCLNM in PTMC. The prevalence of CLNM and NSVCLNM in cN0 PTMC patients were 28.8% (121/420) and 7.9% (33/420), respectively.

In our study, univariate and multivariate analyses were used to evaluate the predictive factors of NSVCLNM. We found that age ≤36 years, male sex, primary tumor of >0.65 cm measured by US, and multifocal lesions identified by US were independent predictive factors of NSVCLNM in cN0 PTMC patients without ETE.

Age is known to be an important prognostic factor for patients with PTMC. Previous studies have reported that younger age is an increased risk of CLNM^[34–36]^ and high-volume (ie, >5 metastatic lymph nodes) CLNM^[37,38]^ in cN0 PTMC patients. In addition, Miyauchi et al have reported that <40 years of age is the only significant risk factor for tumor size enlargement and novel appearance of lymph node metastasis of PTMC during active surveillance.^[39]^ Miyauchi et al have also found that the proportion of appearance of novel lymph node metastasis in PTMC patients <40 years of age is highest (16.1%) after 10 years of observation^[39]^ and that the estimated lifetime probability of PTMC progression was related to the age at diagnosis, being 60.3% for younger (25 years) and 3.5% for older patients (75 years).^[40]^ In our study, patients with NSVCLNM were significantly younger than those without NSVCLNM (37.8 [9.2] vs 44.5 [10.5] years, *P* < .001). We used ROC curve analysis to determine the cutoff age for predicting NSVCLNM which showed that age ≤36 years was significantly associated with an increased risk of NSVCLNM in cN0 PTMC patients.

The incidence of thyroid cancer is >3 times higher in women.^[41]^ However, men tend to more advanced disease diagnosed at an older age, lower disease-free survival, and higher mortality.^[42]^ Previous studies have demonstrated that male sex is a significant risk factor for CLNM^[34–36]^ and high-volume (ie, >5 metastatic lymph nodes) CLNM^[37,38]^ in cN0 PTMC patients. Furthermore, Nixon et al have reported that male sex is the risk factor associated with central neck node recurrence in PTC patients without pCND.^[43]^ In this study, we also found that male sex as a predictive factor for NSVCLNM in cN0 PTMC.

Tumor size is another important factor in TNM staging for PTC, and large tumors are more prone to be aggressive.^[44]^ Noguschi et al found that the recurrence-free survival rate is significantly poorer in the PTMC patients with primary tumors 6 to 10 mm, when compared with patients with primary tumors ≤5 mm (86.0% vs 96.7%, *P* < .0001).^[10]^ This finding strongly suggests that even among PTMC, larger tumors have a worse prognosis compared to smaller ones. Several studies have shown that tumor size was significantly associated with CLNM in PTMC, but the cutoff points were different. Huang et al reported that ultrasonic tumor size >7 mm was the predict factor of high-volume (ie, >5 metastatic lymph nodes) CLNM in cN0 PTMC,^[38]^ whereas Zhang et al^[34]^ and Liu et al^[35]^ reported tumor size >6 mm and >5 mm, were associated with CLNM in cN0 PTMC, respectively. In our study, ultrasonic tumor size in NSVCLNM patients was significantly greater than those without NSVCLNM (0.74 [0.47–1.00] vs 0.60 [0.26–1.00] cm, *P* < .001). ROC curve analysis was used to determine the cutoff point of tumor size for predicting NSVCLNM and found that tumor size >0.65 cm was the strongest predictor of NSVCLNM in cN0 PTMC.

Multifocal disease has been associated with a higher risk of recurrence^[45–48]^ and locoregional metastases^[34,35]^ in PTMC patients in many studies. Multifocal lesions identified by preoperatively US consistently demonstrated a predictive factor of NSVCLNM for cN0 PTMC in our study. Conversely, our ultrasonic results showed that mixed calcifications and blood flow were no significantly related to the presence of NSVCLNM in cN0 PTMC, which was consistent with the previous study reported by Huang et al.^[38]^

To identify the subset of cN0 PTMC patients who are prone to burden of NSVCLNM before operation, a prediction mode based on independent predictive factors was developed. ROC curve analysis was used to determine the cutoff point of predictive value and found that the predictive value of >−2.418 as the strongest predictor of NSCVLNM. The sensitivity and specificity were 69.7% and 84.0%, respectively, which was significantly greater than the efficacy of US in detecting CLNM of PTC.^[49–51]^

However, the present study has several limitations. First, there are inherent features of a nonrandomized retrospective cohort study. Secondly, in our center, ipsilateral prophylactic CND was performed routinely for cN0 ipsilateral PTMCs, whereas bilateral prophylactic CND was performed if the tumors were located in isthmus or bilateral lobes. Thus, the extent of CND in this study included unilateral and bilateral clearance, which may have influence the number and size of metastatic lymph nodes in the central compartment. Thirdly, the patients included in this study, was not very large. Nevertheless, this study has several significant strengths. First, tumor size and multifocality in this study was determined through preoperative US examination. Therefore, all the predictive factors identified in this study can be assessed before operation, which makes our finding a great applicability to the selection of cN0 PTMC patients for immediate surgery. Secondly, an equation has been developed according to the predictive factors, which demonstrated better predictive efficiency than each independent predictive factor. Thirdly, in this study we also confirmed that the 5-year RFS in cN0 PTMC patients with NSVCLNM was significantly poorer, when compared with patients without NSVCLNM.

In conclusion, PTMC patients with NSVCLNM had poorer RFS, when compared with patients without NSVCLNM. In addition, this study demonstrated that age ≤36 years, male sex, ultrasonic tumor sizes of >0.65 cm, and ultrasonic multifocality as strong indicators for NSVCLNM in cN0 PTMC patients without ETE. Based on these data, a mathematical model was proposed to quantitatively predict NSVCLNM. Therefore, when the predictive value was >−2.418 according to the equation (Y = 1.714 × age + 1.361 × sex + 1.639 × tumor size + 0.842 × multifocality −5.196), immediate thyroid surgery including CND rather than active surveillance might be considered for cN0 PTMC patients.

## Author contributions

**Conceptualization:** Jian-Biao Wang.

**Data curation:** Jin-Duo Shou, Fei-Bo Li, Liu-Hong Shi, Liang Zhou.

**Formal analysis:** Jin-Duo Shou, Fei-Bo Li, Liu-Hong Shi, Liang Zhou.

**Methodology:** Jin-Duo Shou, Fei-Bo Li, Jian-Biao Wang.

**Supervision:** Jian-Biao Wang, Lei Xie.

**Writing – original draft:** Jin-Duo Shou, Jian-Biao Wang.

**Writing – review & editing:** Jian-Biao Wang, Lei Xie.

## Abbreviations

Abbreviations: ATA = American Thyroid Association, CI = confidence interval, CLNM = central lymph node metastasis, cN0 = clinically node-negative, cN1 = clinically apparent metastatic disease to nodes, CND = central neck dissection, CT = computed tomography, ETE = extrathyroidal extension, FNA = fine needle aspiration, NPV = negative predictive value, NSVCLNM = non-small-volume central lymph node metastases, OR = odds ratio, pCND = prophylactic central neck dissection, PPV = positive predictive value, PTC = papillary thyroid carcinoma, PTMC = papillary thyroid microcarcinoma, RFS = recurrence-free survival, ROC = receiver-operating characteristic, US = ultrasonography.
